# Crocin Enhanced Functional Recovery after Sciatic Nerve Crush Injury in Rats

**Published:** 2013-01

**Authors:** Esmaeal Tamaddonfard, Amir Abbas Farshid, Elham Ahmadian, Abbas Hamidhoseyni

**Affiliations:** 1 Department of Basic Sciences, Faculty of Veterinary Medicine, Urmia University, Urmia, Iran; 2 Department of Pathobiology, Faculty of Veterinary Medicine, Urmia University, Urmia, Iran; 3 Faculty of Veterinary Medicine, Urmia University, Urmia, Iran; 4 Faculty of Veterinary Medicine, Urmia University, Urmia, Iran

**Keywords:** Crocin, Crush injury, Functional recovery, Rats, Sciatic nerve

## Abstract

***Objective(s): ***Crocin is a constituent of saffron and has many biological functions. The present study aimed to investigate the effects of intraperitoneal (IP) injection of crocin on sciatic nerve regeneration in male Wistar rats.

***Materials and Methods: ***Fifty-four rats were divided into 9 groups: groups 1-4 (intact + normal saline and intact + crocin at doses of 5, 20 and 80 mg/kg, respectively); group 5 (sham surgery + normal saline); groups 6-9 (crush + normal saline and crush + crocin at doses of 5, 20 and 80 mg/kg, respectively). Normal saline and crocin were IP injected for 10 consecutive days after induction of a standard crush injury in left sciatic nerve. Footprints were obtained 1 day before and weekly after induction of nerve injury for evaluation of sciatic functional index (SFI). Blood samples were taken for evaluation of malondialdehyde (MDA) levels. Histopathological changes of sciatic nerve were investigated by light microscopy.

***Results: ***Sciatic nerve crush-injured rats showed SFI values reduction, increased plasma MDA levels and produced Wallerian degeneration in sciatic nerve. Crocin at a dose of 5 mg/kg had no significant effects. At doses of 20 and 80 mg/kg, crocin accelerated the SFI recovery, decreased MDA levels and reduced Wallerian degeneration severity.

***Conclusion: ***The present study suggests that the neuroprotective effects afforded by crocin may be due in part to

reduction of free radicals-induced toxic effects.

## Introduction

The specialized structure of a peripheral nerve bundle is essential to normal sensory, motor and autonomic functions. Injury to peripheral nerves results in temporary or life-long neuronal dysfunction that can subsequently lead to economic or social disability (1, 2). Peripheral nerve transaction or crush leads to an acute myelinoaxonal degerenation in the distal area of the damaged nerve, called Wallerian degeneration. This process is associated with macrophage infiltration, Schwan cell proliferation and axonal re-growth (3). It is known that the free oxygen radical levels of peripheral nerve increase and cause nerve tissue damage due to the tissue destruction after injury (4, 5). 

Crocin is the major yellow pigment in gardenia yellow and saffron, which are extracts of *Gardenia jasminoides* fruits and *Crocus sativus* stigmas, respectively (6, 7). Recent studies have suggested anti-inflammatory, analgesic, antiepileptic, anti-edematos and antioxidant properties of crocin (8-13). Neuroprotective effects of crocin have been reported by some investigators (14-16). Recently, in microtubules extracted from sheep brain, tubulin polymerization and microtubule increasing effects of crocin were reported (17). 

The effects of crocin on functional recovery after peripheral nerve injury, to the best of our knowledge, have not been studied before. In the present study, we investigated the effects of crocin on functional and histological changes following sciatic nerve crush injury. In addition, to identify the mechanism that possibly mediates the effect of crocin on functional recovery after sciatic nerve crush injury, the effect of crocin on plasma level of MDA was also investigated.

## Materials and Methods

***Animals***

Healthy adult male Wistar rats, weighing 250–270 g were used in this study. Rats were maintained in groups of 6 per cage in a light-dark cycle (light on at 07:00 hr) at a controlled ambient temperature (22±0.5°C) with *ad libitum* food and water. Six rats were used for each experiment. All research and animal care procedures were approved by the Veterinary Ethics Committee of the Faculty of Veterinary Medicine of Urmia University and were performed in accordance with the National Institutes of Health Guide for Care and Use of Laboratory Animals.

***Chemicals***

Crocin powder was purchased from Fluka Reidel-deHaen (Buchs SG, Schweiz). Sodium dodecyl sulphate, acetic acid, thiobarbituric acid, n-butanol and pyridine were purchased from Merck Chemical Co. (Darmstadt, Germany). 

***Grouping***

The animals were randomly divided into following groups of six rats. 

Groups 1, 2, 3, 4: Intact groups; received IP injection of normal saline and crocin at doses of 5, 20 and 80 mg/kg, respectively, for 10 consecutive days without sciatic nerve crush injury induction.

Group 5: Sham surgery group; received IP injection of normal saline for 10 consecutive days after surgery without sciatic nerve crush injury induction.

Groups 6, 7, 8, 9: Crush groups; received IP injection of normal saline and crocin at doses of 5, 20 and 80 mg/kg, respectively, after surgery for 10 consecutive days with sciatic nerve crush injury induction. 

The protocol for this study, including doses of crocin was designed according to the previous studies in which the used doses of crocin were 50-200 mg/kg for 5 days, 15 and 30 mg/kg and 5-20 mg/kg for 21 days (18-20). 

***Surgery***

Rats were anesthetized by IP injection of a mixture of ketamine (80 mg/kg) and xylazine (10 mg/kg). The area above the left lower thigh was shaved and sterilized with betadine. A 2-cm incision was made over the lateral aspect of the hind limb, and muscles are separated in order to expose the sciatic nerve. The nerve was crushed at 0.5 cm proximal to its trifurcation point using a small haemostatic forceps, the jaws of which were covered with teflon tubing to provide smooth surface. The nerve was crushed for 60 sec with an estimated pressure of 0.5-1 kg/mm^2^. The crushed zone was approximately 4 mm^2 ^and uniformly transparent for several minutes thereafter. In sham group, the sciatic nerve was exposed but not crushed. The muscle layers were re-approximated using 4/0 chromic gut sutures, and the skin was closed with 3/0 silk sutures. 

***Sciatic functional index***

Evaluation of SFI was done on one day before surgery and on days 7, 14, 21 and 28 following surgery. Rats were held by the chest and their hind feet were pressed down onto a stamp pad soaked with water soluble blue ink. Rats were immediately allowed to walk along a confined walkway 7.5 cm wide by 60 cm long with a dark shelter at the end of the corridor leaving its foot prints on the paper that is cut to the appropriate dimensions and placed on the floor of the corridor. The following measurements were taken from the footprints: (1) distance from the heel to the third toe, the print length (PL); (2) distance from the first to fifth toe, the toe spread (TS); and (3) distance from the second to the fourth toe, the intermediary toe spread (ITS). All three measurements were taken from the experimental (E, undergoing sciatic nerve crush) and normal (N) limbs. Three factors that comprised the SFI were calculated as follows: (1) print length factor (PLF)= (EPL–NPL)/NPL; (2) toe spread factor (TSF)= (EST – NST)/NST; (3) intermediary toe spread factor (ITF)= (EIT–NIT)/NIT. Using these data, the SFI, which indicates the differences between the injured and the intact contralateral paw, was calculated by the following formula derived by Bain *et al* (21): 

SFI= –38.3 [(EPL–NPL)/NPL]+109.5 [(ETS-NTS)/NTS] +13.3[(EIT- NIT)/NIT]-8.8 

The SFI was analyzed as: an SFI equal to -100 indicates significant impairment, whereas an SFI oscillating around 0 is considered to reflect normal function.

***Biochemical assays***

At days 15 and 30 after sciatic nerve lesion, the animals were sedated using diethyl ether and blood samples (0.5 ml) were collected in vials containing heparin by heart puncture. The plasma was separated and kept at -80 ºC until analysis of MDA. MDA, an index of free radical generation/lipid peroxidation, was determined as described by Ohkawa *et al* (22). Briefly, the reaction mixture consisted of 0.2 ml of 8.1% sodium dudecyl sulphate, 1.5 ml of 20% acetic acid (pH 3.5), 1.5 ml of 0.8% aqueous solution of thiobarbituric acid and 0.2 ml of blood plasma. The mixture was made up to 4 ml with distilled water and heated at 95°C for 60 min. After cooling the contents under running tap water, 5 ml of n-butanol and pyridine (15:1 v/v) and 1 ml of distilled water were added. The contents were centrifuged at about 16000 × g for 3 min. The organic layer was separated out and its absorbance was measured at 532 nm. Plasma MDA concentrations were expressed as nmol/ml. 

***Histopathological evaluation***

At day 30 after sciatic nerve injury, the sedated rats were euthanized and distal segment of sciatic nerve was removed and fixed in 10% buffer formal saline. The 10% buffer formal saline fixed sciatic nerves routinely processed for paraffin embedding. Thin sections (4-5 μm) were cut using a microtome and stained with hematoxylin and eosin (H&E) and then examined using a light microscope. The evaluation of the sections was based on the severity of pathological changes on a scale from normal (0) to severe (3) changes.

***Statistical analysis***

All data are presented as mean± SEM. Significance of the SFI and MDA were assessed by two-way analysis of variance (ANOVA) followed by Duncan^,^s test for multiple comparisons. Values for the Wallerian degeneration were analyzed using one-way ANOVA followed by Duncan^,^s test for multiple comparisons. Significance at *P*< 0.05 has been given receptive in all tests. 

## Results

**Figure 1:**
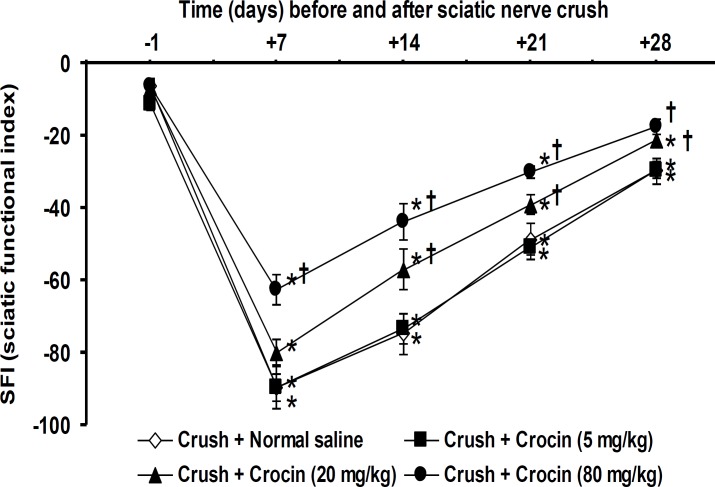
Effect of intraperitoneal (IP) injections of normal saline and crocin on sciatic functional index in sciatic nerve-crushed rats. Data are presented as mean ± SEM (n = 6). *P< 0.05 denotes significant difference *vs* day one before crush. ^†^*P*< 0.05 denotes significant difference *vs* crush + normal saline group

Figure 1 shows the effects of crocin on SFI value changes induced by sciatic nerve crush injury on one day before and on days 7, 14, 21 and 28 after crush. No significant changes on SFI values were observed among groups 1-5 (intact + normal saline group, intact + crocin at doses of 5, 20 and 80 mg/kg groups and sham surgery + normal saline group) on one day before with days 7, 14, 21 and 28 after crush injury. Therefore, the data related to days 7, 14, 21 and 28 after crush injury of groups 1-5 have not been shown in the Figure 1. No significant differences in SFI scores were observed between groups 6 (crush + normal saline group) and 7 (crush + crocin 5mg/kg group) during the four weeks evaluation period. Group 8 (crush + crocin 20 mg/kg group) showed significant (*P*< 0.05) recovery in walking behavior at days 14, 21 and 28 after crush when compared with group 6 (crush + normal saline group). Crocin at a dose of 80 mg/kg (group 9) significantly (*P*< 0.05) recovered SFI values during the four weeks evaluation period (Figure 1). 

**Figure 2:**
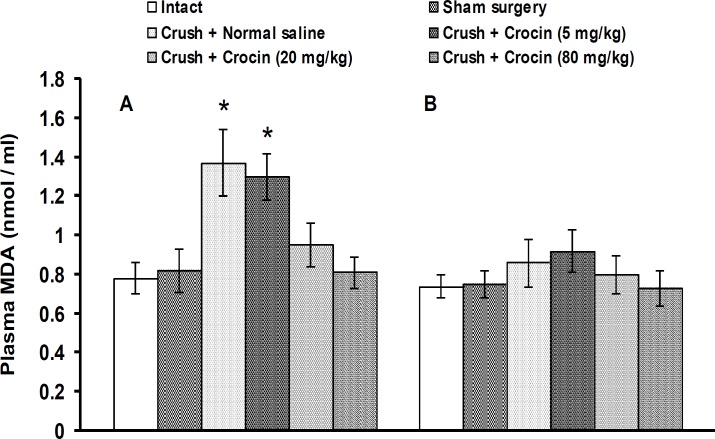
Effect of IP injections of crocin on plasma malondialdehyde (MDA) levels in sciatic nerve-crushed at days 15 (A) and 30 (B) after crush in rats. Data are presented as mean ± SEM (n = 6). *P< 0.05 denotes significant difference *vs* other groups

Figure 2 shows the effects of crocin on plasma MDA level changes induced by sciatic nerve crush injury at days 15 (A) and 30 (B) after crush. No significant changes on MDA levels were observed among groups 1-5 (intact + normal saline group, intact + crocin at doses of 5, 20 and 80 mg/kg groups and sham surgery + normal saline group) at days 15 (0.78 ± 0.08, 0.80 ± 0.09, 0.75 ± 0.08, 0.72 ± 0.010 and 0.82 ± 0.11 nmol/ml plasma, respectively) and 30 (0.74 ± 0.06, 0.73 ± 0.07, 0.79 ± 0.11, 0.74 ± 0.06 and 0.74 ± 0.06 nmol/ml plasma, respectively) after crush. The data related to groups 2-4 have not been shown in figure 2. In group 6 (crush + normal saline and crush group), plasma MDA levels were significantly (*P*< 0.05) increased on day 15, but not on day 30, after crush. Crocin at a dose of 5 mg/kg (group 7) had no significant effect. Groups 8 and 9 (crush + crocin 20 mg/kg and crush + crocin 80 mg/kg groups) showed significant (*P*< 0.05) reductions in plasma MDA levels on day 15 after crush (Figure 2). 

**Figure 3:**
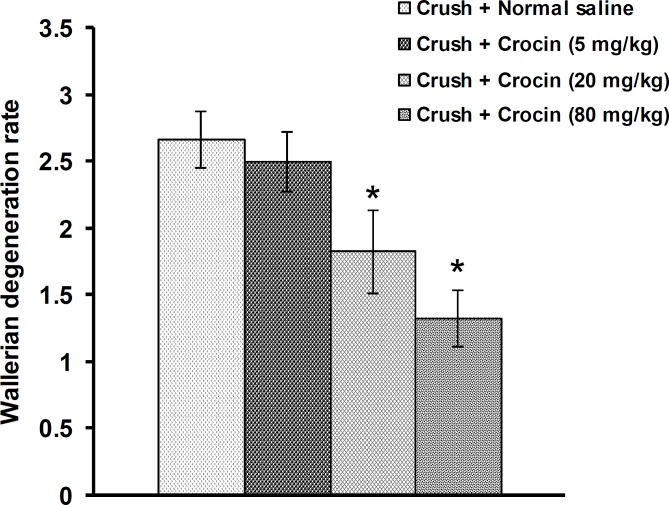
Effect of IP injections of crocin on histopathological changes in distal segment of sciatic nerve induced by crush injury in rats. Data are presented as mean ± SEM (n = 6). *P< 0.05 denotes significant difference *vs*. crush + normal saline group

**Figure 4:**
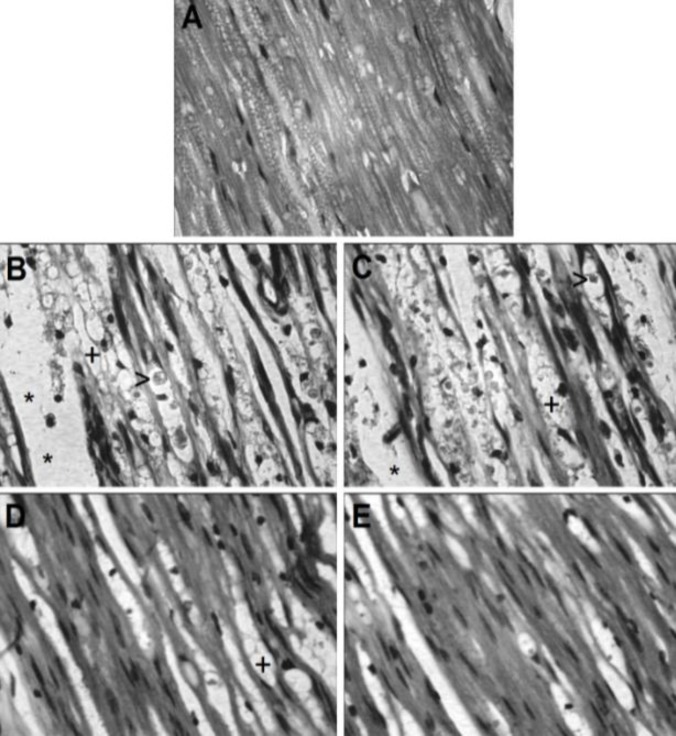
Histological analysis of rat sciatic nerve (A): Intact animals were not submitted to nerve crush and received normal saline (IP). it was observed normal architecture of sciatic nerve. (B and C): Animals submitted to sciatic nerve crush injury and treated with IP injections of normal saline and crocin (5 mg/kg), respectively. It was observed several areas of edema (*), vacuolization (+) and degraded myelin sheath (myelin ellipsoid) (>). (D and E): Sciatic nerve crushed animals received crocin at doses of 20 and 80 mg/kg, respectively. Few areas of edema, vacuolization and degraded myelin (myelin ellipsoid) were observed. (H&E × 400)

Figures 3 and 4 shows the effect of crocin on histopathological changes in the sciatic nerve induced by crush injury. No changes were observed among groups 1-5. The data related to groups 2-5 were not shown in Figures 3 and 4.

In group 1, no histopathological changes were observed (Figures 3 and 4A). In group 6, crush injury produced severe changes in the sciatic nerve, including swelling of myelin sheet, vacuolization and myelin ellipsoids (Figures 3 and 4B). Ip injection of crocin at a dose of 5 mg/kg (group 7) produced no significant effect (Figures 3 and 4C). Crocin at doses of 20 and 80 mg/kg (groups 8 and 9, respectively) alleviated all the histological changes consequent to the crush injury in the sciatic nerve (Figures 3, 4D and 4E). 

## Discussion

In the present study, all animals showed SFI values nearing 0 (normal) before surgery. In sciatic nerve crushed + normal saline treated group, SFI values reached to -89.5 ± 3.8 (severely impaired) on day 7 after crush. From day 14 to day 28 after crush, SFI values gradually returned but not reached to pre-crush values. Several authors reported nearly same results whose studies have also shown normal walking patterns only after the first month of post crush (23, 24). In contrast to these experiments, some authors reported a full recovery at the third and fourth weeks (25, 26). The difference in the motor functional recovery may relate to the pathophysiologic response of peripheral nerves to the magnitude of different crushing loads (27, 28). In this study, plasma MDA levels in group 6 (crush + normal saline group) significantly elevated on day 15, but not on day 30, after crush. This could be accounted for by the tissue^,^s own protective mechanisms. It has been found that serum MDA levels increases on day 7 after crush and then gradually declines on days 14, 21 and 42 after sciatic nerve crush injury in rats (29). Consistently, it has been reported that MDA levels remained high initially and then decreased in ischemia/reperfusion injury of sciatic nerve in rats (30). Moreover, Senoglu *et al* (5) reported an elevation of sciatic nerve tissue MDA levels after sciatic nerve crush injury in rats. MDA, an end product of polyunsaturated fatty acid, is a reliable and commonly used biomarker for assessing lipid peroxidation. Lipid peroxidation is a well-established mechanism of cellular injury and is used as an indicator of oxidative stress in cells and tissues (31). In addition, the results of present study showed swelling of myelin sheath, vacuolization and myelin ellipsoids in the distal side of sciatic side of sciatic nerve after crush injury. Several areas of edema, degraded myelin sheet and mononuclear cell infiltration have been reported to occur in the distal side of sciatic nerve after crush injury in rats (26). 

When an axon is crushed or severed, changes occur in proximal and distal sides of the lesion. Distally, Wallerian degeneration takes place, resulting in axonal degeneration. The myelin sheath of axon detaches, degrades and converts into ellipsoidal segments (32, 33). 

The results of the present study showed that crocin improved the return of SFI values, decreased the elevated MDA levels and reduced Wallerian degeneration after crush injury in sciatic nerve. These indicate that crocin enhanced functional recovery by decreasing the effects of free radicals in inducing the biochemical and histological changes after crush injury in sciatic nerve. Oxidative stress is considered to be one of the main causes of neural damage after injury. It has been reported that antioxidant molecules including catalase, superoxide dismutase and glutathione-S-transferase play an important role in peripheral nerve injury and regeneration (34). Antioxidant agents such as melatonin, N-acetylcysteine and edaravone had promoting effects on sciatic nerve regeneration after nerve crush, sciatic nerve axotomy and grafting and ischemia/reperfusion injury, respectively (35-37). Some medicinal plant extracts including *Achyranthes bidentata* and *Ginkgo biloba* or their active substances such as curcumin from turmeric and ginsenoside Rg1 from ginseng have been reported to exert accelerating effects on peripheral nerve regeneration (38-41). Although, the involvement of saffron and its constituents, crocin and safranal, on sciatic nerve regeneration has not been reported, some researchers have claimed beneficial effects of crocin on nervous system. Saffron and its constituent, crocin protected the brain against excessive oxidation stress in global cerebral ischemia-induced ischemia/reprfusion injury (15, 42). It has been reported that crocin and crocetin blocked the effect of lipopolysaccaride (LPS) on hippocampal cell death by reducing the production of intracellular reactive oxygen species (14). Post-experimental autoimmune encephalomyelitis (EAE) treatment with crocin suppressed inflammatory gene expression in spinal cord, which was accompanied by preserved myelination and axonal density (16). EAE-associated neurobehavioral deficits were also ameliorated by crocin (16). 

## Conclusions

The results demonstrated that sciatic nerve crush injury impaired motor behavior, increased plasma MDA levels and produced Wallerian degeneration in distal segment of nerve. Moreover, treatment with crocin improved motor behavior, recovered plasma MDA levels and prevented histological changes in sciatic nerve. The neuroprotective effect of crocin may be mediated through its antioxidant effect.
